# Bacteriological and Physical Quality of Locally Packaged Drinking Water in Kampala, Uganda

**DOI:** 10.1155/2015/942928

**Published:** 2015-10-05

**Authors:** Abdullah Ali Halage, Charles Ssemugabo, David K. Ssemwanga, David Musoke, Richard K. Mugambe, David Guwatudde, John C. Ssempebwa

**Affiliations:** ^1^Department of Disease Control and Environmental Health, School of Public Health, College of Health Sciences, Makerere University, P.O. Box 7072, Kampala, Uganda; ^2^Department of Epidemiology and Biostatistics, School of Public Health, College of Health Science, Makerere University, P.O. Box 7072, Kampala, Uganda

## Abstract

*Objective*. To assess the bacteriological and physical quality of locally packaged drinking water sold for public consumption. *Methods*. This was cross-sectional study where a total of 60 samples of bottled water from 10 brands and 30 samples of sachet water from 15 brands purchased randomly were analyzed for bacteriological contamination (total coliform and faecal coliform per 100 mL) using membrane filtrate method and reported in terms of cfu/100 mL. *Results*. Both bottled water and sachet water were not contaminated with faecal coliform. Majority (70%, 21/30) of the sachet water analyzed exceeded acceptable limits of 0 total coliforms per 100 mL set by WHO and the national drinking water standards. The physical quality (turbidity and pH) of all the packaged water brands analyzed was within the acceptable limits. There was statistically significant difference between the median count of total coliform in both sachet water and bottled water brands (*U*(24) = 37.0, *p* = 0.027). *Conclusion*. Both bottled water and sachet water were not contaminated with faecal coliforms; majority of sachet water was contaminated with total coliform above acceptable limits. Government and other stakeholders should consider intensifying surveillance activities and enforcing strict hygienic measures in this rapidly expanding industry to improve packaged water quality.

## 1. Introduction

Packaged water is any potable water that is manufactured or processed for sale which is sealed in food-grade bottles, sachets, or other containers and intended for human consumption [1]. Sale of packaged water has exploded all over the world in recent years, largely as a result of public perception that it is safe, tastes better, and has a better quality compared to raw tap water [2–5]. The increment in consumption globally could also be due to a result of an increase in per capita use as well as population growth [6, 7]. Even in countries where tap water quality is considered excellent, demand was so high, making packaged water the fastest growing product of the nonalcoholic beverage market worldwide [8, 9]. The above situation is not any different in the study area, Kampala, and all over Uganda where water packaged in bottles and polythene bags has become a very common consumer product most especially in the urban centres. The high demand may attract substandard products and counterfeits into the market.

With this increase in consumption of packaged water, there is a possibility of producing products that are not fit for human consumption because of monetary interests [10]. Access to safe drinking water is still one of the major challenges of the 21st century [11]. Unsafe water is a global public health threat, placing persons at risk for a host of diarrhoeal diseases as well as chemical intoxication [12]. Although disease outbreaks due to contaminated packaged water are not common, any possible contamination may lead to widespread epidemic because of the high demand and coverage [13]. Therefore access to an adequate and safe water supply is very important and can result in significant benefits to health [14]. Packaged water has ever been implicated as a source of outbreaks of cholera and typhoid fever as well as traveler's disease in countries such as Portugal and Spain [1, 15–17]. Several studies have shown that packaged water can be contaminated with bacteria at various stages of production [18–23]. Under improper or prolonged storage of bottled water, bacteria can grow to levels that may be harmful to human health [1, 4, 5].

In Kampala, production and consumption of packaged water are increasing at a very high rate. However, continuous surveillance of its quality at retail premises is not being carried out. This may lead to consumption of poor quality packaged water. There has also been a growing concern about the microbiological quality of the products. Therefore, this study assessed the physical and bacteriological quality of locally packaged water sold for public consumption in Kampala, Uganda.

## 2. Materials and Methods

### 2.1. Study Setting and Design

This was a cross-sectional study carried out in Kampala, the capital city of Uganda. Kampala is divided administratively into 5 divisions, namely, Central division, Kawempe division, Makindye division, Nakawa division, and Lubaga division. It covers a total area of 195 square kilometers with a population of 1,516,210 [24]. Available sources of drinking water in the city include spring water, piped water, wells, boreholes, and lake. The three major causes of morbidity and mortality in Kampala are malaria, respiratory infections, and diarrhoeal diseases. There are over 20 bottled water brands in Uganda and around 14 of them are in Kampala. However, the common brands on market are 10. The number of sachet water manufacturing premises is not known since most of them operate illegally.

### 2.2. Data Collection and Laboratory Analysis

#### 2.2.1. Selection of the Divisions and Parishes

Three of the five municipalities in the city, namely, Lubaga, Makindye, and Kawempe, were randomly selected for involvement in the study. Balloting method with replacement was used. One parish from each municipality was chosen at random using the same method. Main trading centres of the selected parishes were purposively selected because it is where most of the packaged water brands sold were likely to be found. The selected trading centres in each municipality were Bwaise in Kawempe, Kasubi in Lubaga, and Kabalagala in Makindye municipality.

#### 2.2.2. Sampling of Packaged Water

A total of 90 samples (60 bottled and 30 sachets) of all the common brands of packaged water available on market during the month of January were collected for this study. A total of 10 brands of bottled water and 15 brands of sachet water were sampled. All samples were purchased randomly from several retail outlets and supermarkets in the selected trading centres of Kampala. At the retail outlets, duplicate samples (two) bottles of water of each brand and of the same batch were procured from each of the three selected trading centres, thus 20 samples from each municipality giving a total of 60 bottled samples. All the fifteen sachet water brands available on market were sampled. On average, five different brands of sachet water were sampled in duplicate from each of the three trading centres giving a total of 15 brands and 30 samples of sachet water.

All the samples were retained in their original sealed containers and clearly marked for identification with letters from A to J for bottled water and from K to Y for sachet water samples. They were transported to School of Public Health Laboratory, Makerere University, at Kasangati in Kampala in a sample carrier containing ice packs and analysed within 2 to 4 hours of collection. Then 25% of the samples, a blank sample (a negative control) and spiked sample (a positive control), were sent to National Water and Sewerage Corporation (NWSC) laboratory in Kampala for analysis so as to compare with the analysis findings.

#### 2.2.3. Water Sample Analysis in the Laboratory

The membrane filtration method for bacteriological analysis with M-Lauryl Sulphate Broth as culture medium was used. 100 mL of water from each sample was filtrated using a membrane filter and then incubated at 44.5°C and 37.5°C for 18 hours for faecal and total coliforms, respectively. The samples were analysed in duplicate and the average was recorded. These temperatures are known to be optimal for the growth of these bacteria. Turbidity was measured using turbidity tubes and reported in NTU and pH using Wagtech pH meter. The equipment was calibrated before use. The analysis for total coliform and faecal coliform organisms per 100 mL was reported in terms of cfu/100 mL. Glassware or materials used in this experiment were washed with distilled water and then autoclaved at 121°C for 15 minutes to ensure sterility. The bacteriological and physical quality of the packaged water was determined by comparing it with the national and WHO standards for packaged water which requires water samples of good quality to be with 0 coliforms per 100 mL of water and turbidity of <5 NTU and pH of 6.5–8.5, respectively.

#### 2.2.4. Data Analysis and Management

Data was entered using SPSS version 17.0 computer software for analysis. Data was analysed using both descriptive and inferential statistics. For the descriptive statistics, frequencies and cross tabulations were generated. The main outcome was bacteriological quality of the packaged water (presence or absence of either total coliform or faecal coliform). Other outcomes were turbidity level (within the acceptable limits or not) and pH (within the acceptable limits or not). Mann-Whitney *U* test was used to compare median total coliform count of bottled water to that of sachet water. Wilcoxon signed-rank test was used to test whether the mean of the packaged water brands was statistically different from the required standard of zero total coliform forming units per 100 mL. Fisher's exact test was used to compare the proportion of bottled water with total coliform to that of sachet water. The strength of association was determined using odds ratio and *p* values at 95% level of confidence. The test for normality of the data used was Shapiro-Wilk test.

### 2.3. Quality Control

To ensure quality, water samples were transported and analysed within 2–4 hours. Additionally, samples were analysed in duplicate using standard methods. Equipment was calibrated before use, a blank sample and a spiked sample were included in the analysis, and a reference laboratory (NWSC) was used for validation of the findings.

### 2.4. Ethical Considerations

Approval to conduct the study was obtained from Makerere University School of Public Health Higher Degree Research and Ethical Committee. Permission was also obtained from Kampala Capital City Authority.

## 3. Results

### 3.1. Bacteriological and Physical Quality of Packaged Drinking Water on Sale

The median total coliform count of sachet and bottled water samples was 3.0 and 0.0 per 100 mL of water, respectively. The median pH and turbidity of both bottled water and sachet water were within the acceptable range. All the samples (both bottled water and sachet water) showed negative growth for faecal coliform as shown in Table 1.

### 3.2. Association between Total Coliform and Type of Packaged Water

Using Wilcoxon signed-rank test, the median total coliform count in sachet water brands was found to be significantly above the acceptable level of zero (*Z* = −2.94, *p* value = 0.003) but that of bottled water brands was not (*Z* = −1.633, *p* value = 0.102). Therefore, sachet water is contaminated with total coliform.

Mann-Whitney *U* test showed a statistically significant difference between the median count of total coliform in both sachet water and bottled water brands (*U*(24) = 37.0, *p* = 0.027).

Majority (70%, 21/30) of the sachet water samples and a few (15%, 9/60) of bottled water samples analysed showed positive growth for total coliform above the acceptable levels of 0 cfu/100 mL of water set by WHO and national standards. Most (73.3%, 11/15) of sachet water and a few (30%, 3/10) of bottled water brands had total coliform count above the acceptable limit of 0 cfu/100 mL as shown in Table 2.

The odds of sachet water being likely to be contaminated with total coliform were 13 times higher when compared to bottled water (OR = 13.2, CI: 4.12–43.58) as shown in Table 3.

## 4. Discussion

The physical and bacteriological state of packaged water is a very important aspect that should be observed by all the packaging companies. Bottled water is generally of good quality for drinking, but if it is not properly protected during bottling and transit, it could be contaminated [16]. As per the national and WHO standards, packaged water should have turbidity of less than 5 NTU, pH range of 6.5–8.5, and total coliform and faecal coliform of 0.00 per 100 mL. As a measure for safety, indicator organisms of coliforms are investigated and their presence generally indicates that the water may contain disease causing agents. Coliforms are in two categories: total (all) coliforms count which indicates general contamination and faecal coliform count which indicates faecal contamination and is a significant indicator of pollution.

The physical properties of packaged water showed that pH and turbidity were within the permissible limits which indicate that packaged water was suitable for consumption. These results are similar to studies conducted in Delhi, India; Alexandria, Egypt; and Kano state, Nigeria [25–27]. However, these results contradict results from studies carried out in Ghana and Nigeria which showed pH values for sachet water ranging from 5.4 to 7.6 [28, 29] and in Amhara, Ethiopia, from 5.3 to 5.5 [30] which implies that some of the packaged water was not suitable for human consumption. Another study carried out in Georgia, United States, showed that sachet ice water had 95 and 3 samples with pH and turbidity outside the acceptable levels [31].

The study findings showed that some bottled water samples and most of the sachet water samples had total coliform above the acceptable levels for human consumption and the deference was statistically significant between the 2 categories (*p* = 0.027). These findings to a greater extent concur with the findings of other studies [9, 17–21, 32–35]. However, they are similar to findings from a study carried out in Lahore, Pakistan [36], where 3 out of the 10 bottled and 4 out of the 15 sachet water brands tested did not meet WHO and national standards of 0 cfu per 100 m, and other studies in sub-Saharan Africa, where detectable total coliforms were found in packaged water [5, 37]. Correspondingly, the proportion of sachet water contaminated with total coliforms at 73.3% was higher than that in a study conducted in Accra, Ghana, where only 18% of the samples exceeded this level [9]. Unlike these studies, in Nigeria, all sachet water samples had total coliforms [38]. The rate of contamination varied according to the type of packaged water. The rate of total coliform contamination in sachet water was higher than that in bottled water. This finding concurs with those of studies conducted in Delhi and Jaipur, India; Dar es Salaam, Tanzania; and Kumasi, Ghana [18, 20, 25, 32]. Furthermore, none of the samples of both bottled water and sachet water were positive for faecal coliform and exceeded the acceptable turbidity level and pH range. This finding is consistent with the finding of other studies conducted by [9, 13, 32, 39] who found out no microbial indicators of faecal contamination in packaged water.

The presence of total coliform in packaged water can be linked to a number of factors such as the raw water source used, hygienic practices observed during production, improper storage in unhygienic and high temperature conditions, and lack of protective measures due to the common treatment methods used (ozonation and ultraviolet light) against bacterial regrowth [40, 41]. In view of this, the possibility of regrowth of microorganisms is greatly increased considering that the temperature in Kampala may be as high as 28°C. Studies conducted in the United Arab Emirates and United States of America demonstrated that organisms multiply more easily between 25°C and 37°C [21, 42]. Another study carried out in Freetown, Sierra Leone, showed that increase in concentration of total coliforms may be due to growth of microorganisms already present within the packaged water [5]. The findings suggest need for the government and other stakeholders to intensify surveillance activities and enforce strict hygienic measures in this rapidly expanding industry to improve water quality. The source and treatment process of sachet water therefore need further investigation.

Some of the possible reasons why sachet water is more likely to be contaminated than bottled water could be attributed to better hygienic practices observed in the bottled water industry compared to sachet water industry. These include use of protective sealed caps on bottles, improved and hygienic filling system, source of water, time between production and sale, and use of nonreturnable plastic containers [5, 18, 20]. In Kampala the most common source of water used in sachet water is springs which are most likely to be contaminated [43]. There may also be a possibility that sachet water was contaminated before packaging as observed by [23, 44]. Other reasons could be due to plastic sachets that can easily be punctured and leak compared to plastic bottles; sachet water is most likely to be hand filled with water of suspect quality under unhygienic conditions and sold cheaply; it is much easier to reseal a plastic sachet than to mend the broken seal of water bottle for producing counterfeits. Lastly, sachet water producers are operating illegally; therefore, they are not registered and being monitored.

Despite the presence of total coliforms in some packaged water samples, it may be safer than other alternative drinking water sources. Presence of faecal coliform is the most important indicator for water contamination. During the typhoid outbreak in Kampala, majority of the water sources tested were found to be contaminated with faecal coliforms [45]. In addition, spring water sources in Kampala were found to be contaminated with faecal coliform [43]. With only 85% of the population in Kampala having access to improved sources of drinking water [46], many consumers may resort to alternative sources, including sachet water, wells, and springs, among which sachet water may be the safest. Therefore, there is need to balance protection of public health and access to drinking water as emphasized in some studies conducted in Sierra Leone and Ghana [5, 47]. These findings suggest the need for the government and other stakeholders to intensify surveillance activities and enforce strict hygienic measures to packaged water industries to improve water quality, The source, treatment process, and stage (retail outlet or manufacturing premises) of packaged water contamination should be investigated.

*Study Limitations*. In this study we only analysed for turbidity and pH as indicators for physical quality.

## 5. Conclusions and Recommendations

It can be concluded from the results of this study that both bottled water and sachet water sold at retail outlets were free from faecal contamination. Majority of sachet water samples were contaminated with total coliform above acceptable limits for human consumption. Findings suggest need for the government and other stakeholders to intensify surveillance activities and enforce strict hygienic measures to packaged water industries and retail outlets to improve water quality. The source, treatment process, and stage (retail outlet or manufacturing premises) of packaged water contamination should be investigated.

## Figures and Tables

**Table 1:** Descriptive statistics of the total coliform count, pH, and turbidity in packaged water brands.

Parameters	Median	Std. deviation	Range	Max.	Min.	Standards	
*Bottled water*						WHO	National
Total coliform	0.0	3.44	11	11	0.0	0	0
Faecal coliform	0.0						
pH	7.2	0.32	1.1	7.8	6.8	<8.0	6.5–8.5

Turbidity	0.0	0.0	0.0	0.0	0.0	<5	<5

*Sachet water*							
Total coliform	3.0	18.9	65.0	65.0	0.0	0	0
Faecal coliform	0.0	0.0	0.0	0.0	0.0	0	0
pH	7.0	0.33	1.0	7.6	6.7	<8.5	6.5–8.5

Turbidity	0.0	0.0	0.0	0.0	0.0	<5	<5

**Table 2:** Proportion of packaged water samples and brands exceeding permitted total coliform, faecal coliform, pH, and turbidity limits.

Packaged water parameters (cfu/100 mL)	Bottled (*n* = 60)	Sachet (*n* = 30)	WHO standards per 100 mL	National standards per 100 mL
	Number (%)	Number (%)		
*Total coliform*			0	0
Exceeding	09 (15.0)	21 (70.0)		
Not exceeding	51 (85.0)	09 (30.0)		
*Brands exceeding permitted total coliform counts *				
Exceeding	DEG	LOUV		
Not exceeding	ABCFHIJ	KMNPQRSTWXY		
*Faecal coliform *			0	0
Exceeding	00 (00)	00 (00)		
Not exceeding	60 (100)	30 (100)		
*Turbidity*				
Exceeding	00 (00)	00 (00)	<5	<5
Not exceeding	60 (100)	30 (100)		
*pH*			<8.5	6.5–8.5
Exceeding	00 (00)	00 (00)		
Not exceeding	60 (100)	30 (100)		

**Table 3:** Association between packaging materials for drinking water and total coliform occurrence in packaged water.

Variable	Bottled water (*n* = 60)	Sachet water (*n* = 30)	Odds ratio (Fisher's exact test)	95% CI
	Number (%)	Number (%)		
*Total coliform*				
Present	09 (15.0)	21 (70.0)	13.2	4.12–43.58
Absent	51 (85.0)	09 (30.0)		
Total	60 (100)	30 (100)		
